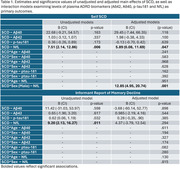# Subjective Cognitive Decline is associated with blood‐plasma Neurofilament Light in cognitively normal older adults

**DOI:** 10.1002/alz.087052

**Published:** 2025-01-09

**Authors:** Silvia Chapman, Stephanie Cosentino, Rachel L. Nosheny, Mathieu Herman, Adam M. Brickman, Sabrina Simoes

**Affiliations:** ^1^ Columbia University Irving Medical Center, New York, NY USA; ^2^ University of California, San Francisco, San Francisco, CA USA

## Abstract

**Background:**

Novel plasma markers are increasingly accepted as indicators of Alzheimer’s disease and related dementia (ADRD) pathophysiology. The extent to which these markers map with clinical symptoms of disease remains unclear. A proposed early clinical symptom of ADRD is subjective cognitive decline (SCD), the experience that cognition has declined despite normal performance on objective assessment. Mapping the association between ADRD plasma biomarker concentrations and SCD will shed light on the links between pathophysiology and symptomology in the earliest stages of disease. This pilot study examines how SCD (self and informant based) associates with plasma p‐tau181, Aβ42, Aβ40 and neurofilament light chain (NfL) concentrations.

**Method:**

Forty‐four (mean aged=68 years) cognitively unimpaired individuals and 41 informants were recruited from the Columbia University Alzheimer's Disease Research Center. Levels of plasma ADRD biomarkers (Aß42, Aß40, p‐tau181 and NfL) were measured on the Simoa HD‐X platform. SCD was measured with two dichotomous variables indicating whether there had been a decline in memory relative to previous ability reported by the participant and their informant. Eight unadjusted linear regression models examined self and informant SCD as predictors of each plasma marker. Models were then adjusted for relevant covariates, and interactions with age and sex were considered.

**Result:**

As reported in Table 1, unadjusted models showed that self‐reported SCD was associated with higher NfL. Informant‐reported SCD was also associated with higher plasma NfL as well as higher p‐tau181. Effect sizes were reduced after adjustment and only self‐reported SCD remained associated with higher NfL. Male sex was associated with greater levels of NfL in those who had SCD.

**Conclusion:**

SCD had stronger associations with a marker of neurodegeneration than amyloid or tau burden. Findings are in line with previous studies showing that NfL has a close relationship with clinical impairments such as cognitive decline. Sample size and type of assay utilized in this study could have limited our ability to see associations with tau and amyloid burden. Futures studies should examine how the combination of plasma markers and early clinical symptoms can aid the predictive utility of plasma markers for onset and progression of clinical symptoms.